# Assessment of reproductive health and violence against women among displaced Syrians in Lebanon

**DOI:** 10.1186/1472-6874-14-25

**Published:** 2014-02-20

**Authors:** Amelia Reese Masterson, Jinan Usta, Jhumka Gupta, Adrienne S Ettinger

**Affiliations:** 1 Department of Chronic Disease Epidemiology, Yale School of Public Health, 60 College Street, New Haven, CT 06510, USA; 2 Department of Family Medicine, American University of Beirut Medical Center, Riad El Solh, Beirut 1107 2020, Lebanon; 3 Yale Center for Perinatal, Pediatric & Environmental Epidemiology, 1 Church Street, 6th floor, New Haven, CT 06510, USA

**Keywords:** Violence against women, Stress, Refugee, Reproductive health, Syria

## Abstract

**Background:**

The current conflict in Syria continues to displace thousands to neighboring countries, including Lebanon. Information is needed to provide adequate health and related services particularly to women in this displaced population.

**Methods:**

We conducted a needs assessment in Lebanon (June-August 2012), administering a cross-sectional survey in six health clinics. Information was collected on reproductive and general health status, conflict violence, stress, and help-seeking behaviors of displaced Syrian women. Bivariate and multivariate analyses were conducted to examine associations between exposure to conflict violence, stress, and reproductive health outcomes.

**Results:**

We interviewed 452 Syrian refugee women ages 18–45 who had been in Lebanon for an average of 5.1 (± 3.7) months. Reported gynecologic conditions were common, including: menstrual irregularity, 53.5%; severe pelvic pain, 51.6%; and reproductive tract infections, 53.3%. Among the pregnancy subset (n = 74), 39.5% of currently pregnant women experienced complications and 36.8% of those who completed pregnancies experienced delivery/abortion complications. Adverse birth outcomes included: low birthweight, 10.5%; preterm delivery, 26.5%; and infant mortality, 2.9%. Of women who experienced conflict-related violence (30.8%) and non-partner sexual violence (3.1%), the majority did not seek medical care (64.6%). Conflict violence and stress score was significantly associated with reported gynecologic conditions, and stress score was found to mediate the relationship between exposure to conflict violence and self-rated health.

**Conclusions:**

This study contributes to the understanding of experience of conflict violence among women, stress, and reproductive health needs. Findings demonstrate the need for better targeting of reproductive health services in refugee settings, as well as referral to psychosocial services for survivors of violence.

## Background

The current humanitarian crisis in Syria, which began during spring 2011, continues to displace Syrians across the country [1-4]. The number of displaced Syrians who have fled to nearby Lebanon is now over 700,000 (October 2013) up from 48,000 just over a year ago (August 2012) [4]. About 24% of these refugees are women between the ages of 18–59 [4]. The mountainous border region of North Lebanon and the Bekaa Valley along Lebanon’s eastern border have received the majority of refugees (34% and 29%, respectively) [4].

North Lebanon and the Bekaa Valley are underserved regions with nearly 53% and 30%, respectively, living below the poverty line in 2008 [5]. Additionally, based on a multidimensional poverty index, 49% of households in North Lebanon and 45% in the Bekaa Valley were deprived of health services in 2004 [6]. With the influx of Syrian refugees, resources in these two regions are now even more strained.

Research shows that women may be more susceptible to poor reproductive health outcomes and violence, including sexual and gender-based violence (SGBV), in conflict settings and refugee crises [7-13]. SGBV has been found to increase surrounding armed conflict, both as a weapon of war and as intimate partner violence, particularly in refugee settings, and reported SGBV prevalence varies greatly [10,12-15]. Violence against women (VAW) can have direct health consequences such as injury, psychological trauma or stress, and gynecologic problems [8,16]. Additionally, stress may be a mediator between exposure to violence in conflict settings and a variety of poor health outcomes among women [15-17]. Numerous anecdotal reports describe VAW related to the Syrian conflict, but assessment of this critical issue is scarce [18-21].

The relationship between conflict violence and reproductive health outcomes may be affected by a variety of factors, including: stress or anxiety [17,22]; socio-demographic indicators such as age, education, and marital status [22,23]; location in Lebanon, urban vs. rural status, and months as a refugee, which could be related to exposure to violence for geopolitical reasons and may also pertain to certain reproductive health outcomes based on healthcare access or local practices [23]; food insecurity resulting from violence and displacement, which may contribute to poor reproductive health outcomes through behavioral, nutritional, or mental health pathways [8,16]; cigarette smoking [16]; anemia [10]; and hypertension [24]. We assessed the potential confounding effects of such variables and adjusted our analyses accordingly.

The overall goal of this study was to increase understanding of reproductive health concerns in a conflict setting by assessing the experiences of displaced women in Lebanon who have recently fled from the conflict in Syria. This aligns with research priorities set by the Interagency-Working Group (IAWG) on Reproductive Health in Crisis and adds to the existing literature on violent conflict, SGBV, and refugee reproductive health by extending current research to a Middle Eastern setting that is experiencing ongoing conflict [25].

## Methods

### Study participants and location

This needs assessment was conducted in Lebanon between June and August 2012, as refugee numbers were escalating in Lebanon one year after the Syrian conflict began. The study was carried out at three primary health clinics located in each of two regions of Lebanon: North Lebanon and the Bekaa Valley under the auspices and at the request of the United Nations Population Fund (UNFPA) Beirut, Lebanon office. Clinics were selected based on the number of displaced Syrian women attending per month (at least 100) and the provision of reproductive health services. In order to minimize bias related to clinic selection, a mix of clinics supported by government and non-government sources was chosen with all receiving some type of support from UNFPA. Three clinics were supported by a private foundation, two clinics were jointly run by the Lebanese Ministry of Public Health (MOPH) and a private foundation, and one clinic was run solely by MOPH. Since none of the local clinics had their own IRBS, ethics approval was sought and obtained from the Human Subjects Committee at Yale School of Public Health (YSPH) and UNFPA/Lebanon using standard procedures for written approval of study protocols and all ethical standards for human subjects research were adhered to throughout the study period.

A cross-sectional survey was carried out in the six primary healthcare clinics. We used a proportional sampling method, based on the number of Syrian women attending each clinic during the month prior to the study, to recruit at least 400 displaced Syrian women. All female, displaced Syrians presenting to these six clinics within the month of July 2012 were approached, screened for eligibility, and asked if they would like to participate until the target number was reached. Eligibility criteria included: ability to speak Arabic, identity as a Syrian national, arrival in Lebanon since the conflict in Syria began in March 2011, and age between 18 and 45 (inclusive). Once screened, women were escorted into a private room where an IRB-approved consent form was explained and signed prior to questionnaire administration.

### Data collection

The interviewer-administered questionnaire was adapted from the “Gender-based Violence Tools Manual For Assessment & Program Design, Monitoring & Evaluation in Conflict-Affected Settings” and the “Reproductive Health Assessment Toolkit for Conflict-Affected Women” [26,27]. The questionnaire was designed in English, discussed with the stakeholders, translated into Arabic and pilot tested among Syrians in Lebanon, then administered in Arabic by trained female research assistants from the area. It addressed the following topics: 1) individual and displacement characteristics; 2) general health status; 3) reproductive history and current status; 4) pregnancy; 5) exposure to violence during the conflict, including sexual violence; and 6) help-seeking behaviors and stress. Of the 489 Syrian women approached to participate, nine did not meet eligibility criteria, 28 declined, and 452 (92.4%) completed the interview. Those who declined to participate cited the following reasons: not interested (28.6%), husband/family member would not allow (25.0%), ill/incapacitated (21.4%), fear (17.9%), and other reason or missing (7.1%). At the end of the interview, participants received a UNFPA “dignity kit” containing basic sanitation supplies and clothing to compensate for their time and were also given telephone numbers for agencies providing psychosocial and other resources for survivors of violence.

### Data analysis

Bivariate associations were estimated using Pearson correlations and *X*^2^ test. Risk factors associated with any of the outcomes of interest, and covariates associated with both risk factors and outcomes, at the level of p < 0.05 were retained in multivariate models. Multivariate logistic regression was used to examine relationships between independent variables (exposure to conflict violence and stress score) and health outcomes (gynecologic outcomes, self-rated health, and reproductive health services access).

A positive response (“1–2 times” or “frequently”) to any of the indicators of violence from an armed person since the conflict began (including: being slapped or hit; choked; beaten or kicked; threatened with a weapon; shot at or stabbed; detained against will; intentionally deprived of food, water, or sleep; emotional abuse or humiliation; deprived of money; or subjected to improper sexual behavior) was considered “exposure to violence,” which was coded as a binary variable. Stress was assessed using a 6-question subscale used previously by UNFPA among conflict-affected populations in Lebanon, with the addition of a question about child beating as an indicator of stress based on qualitative findings among Syrian women. Questions covered the following: feeling constantly tense, sick or tired, worried or concerned, irritable or in a bad mood, suffering from loss of sleep or sleep disorders, reduced ability to complete normal tasks, and beating or taking anger out on children. Principle-component analysis was conducted to determine if this subscale accurately reflected the construct of stress, and to create a stress score variable based on participant responses. The following variables were examined as potential confounders in the relationship between violence or stress and reproductive health outcomes using bivariate analysis; biologically plausible potential confounders were controlled for in multivariate analyses: age, education, marital status, region in Lebanon, clinic and clinic type (government funded or not), place of origin (urban versus rural), months in Lebanon, food insecurity indicators, cigarette smoking, anemia, and hypertension. Data were analyzed using Statistical Analysis Software (SAS) version 9.2, and principle-component analysis was carried out in IBM SPSS Statistics 21.

## Results

### Descriptive statistics and displacement characteristics

Of the 452 displaced Syrian women interviewed, 251 lived in North Lebanon and 201 in the Bekaa Valley (Table 1). Demographic characteristics were similar across regions with several exceptions: those in the Bekaa Valley were slightly younger (p = 0.03) and less likely to have been married (p = 0.01), more women were living in formal housing in North Lebanon (91.6%) than in the Bekaa Valley (80.0%) (p = 0.0004), women in North Lebanon had been in Lebanon longer on average (6 months) than those in the Bekaa Valley (4.5 months) (p = 0.001), and those in the Bekaa Valley were more likely to receive humanitarian services than those in North Lebanon (88.6% and 62.9%, respectively) (p < 0.0001). Of relevance to reproductive health, women reported lack of access to amenities for basic hygiene including: piped drinking water (31.9%), feminine hygiene products (27.7%), washing water (25.9%), soap (26.3%), and bathing facilities (20.8%). Participants reported crowded conditions with 12.8% living with more than five children and five adults. Additionally, the majority responded positively to all three indicators of food insecurity. With regard to general health, most participants rated their overall health as acceptable (38.9%), with 17.7% rating their health as poor or very poor. The majority (79.7%) reported never smoking cigarettes or a water pipe (85.2%). Anemia was the most commonly reported condition with an overall prevalence of 27.4%. Medication use was reported by 39.4%, most commonly for: cardiovascular conditions (6.9%), mental health conditions (5.3%), and gynecologic infections (4.4%).

**Table 1:** Individual characteristics, displacement characteristics, and general health status of 452 Syrian refugee women in Lebanon*

	**N (%) or Mean (±SD)**
**Individual characteristics**	
Region of current residence:	
North Lebanon	251 (55.5)
Bekaa Valley	201 (44.5)
Age:	
18-24	117 (25.9)
25-34	194 (42.9)
35-45	138 (30.5)
Education:	
No education	63 (13.9)
Less than high school	129 (28.5)
High school	152 (33.6)
Greater than high school	104 (23.0)
Marital Status:	
Married	381 (84.3)
Widowed	11 (2.4)
Divorced/separated	6 (1.3)
Never married	54 (12.0)
Consanguineous marriage†	172 (38.1)
Age at first marriage†	19.0 ± 4.0
Currently employed	11 (2.4)
Primary source (s) of income:	
No income	153 (33.9)
Husband	171 (37.8)
Family	61 (13.5)
Self	14 (3.1)
Charity/assistance	98 (21.7)
**Displacement characteristics**	
From a city in Syria (not a village)	221 (48.9)
Reasons for leaving Syria:	
Security concerns/fear	445 (98.5)
Lack of daily necessities	306 (67.7)
Lack of healthcare	279 (61.7)
Other (financial need, home destroyed)	13 (2.9)
Living situation in Lebanon:	
Residing in informal housing (tent, shop, school, etc.)	61 (13.5)
Months in current place of residence	4.6 ± 3.6
Months in Lebanon	5.1 ± 3.7
No. of children (<18) in residence	3.8 ± 2.8
No. of adults (>18) in residence	3.7 ± 3.7
Food insecurity:	
Worry about having enough food (sometimes/often)	284 (62.8)
Eat non-preferred food (sometimes/often)	264 (58.4)
Skip meals (sometimes/often)	249 (55.1)
General health status	
Self-rated overall health:	37 (8.2)
Excellent	157 (34.7)
Good	176 (38.9)
Acceptable/fair	64 (14.2)
Poor	16 (3.5)
Very poor	
Cigarette smoker (some days/every day)	90 (19.9)
Chronic conditions:	
Anemia	124 (27.4)
Hypertension	55 (12.2)
Diabetes	14 (3.1)
Others‡	120 (26.6)
Currently on medication for any condition	178 (39.4)

*Numbers may not sum to total due to missing data.

†Denominator is ever-married women (n = 398).

‡Others: musculoskeletal issues, cardiovascular issues, abdominal issues, mental health and psychosomatic issues, vaginal infections, and urinary tract infections.

### Reproductive and sexual health

The majority of women reported gynecologic problems during the conflict (Table 2), including: menstrual irregularity (53.5%), symptoms of reproductive tract infection (53.3%), severe pelvic pain or dysmenorrhea (51.6%), and 37.8% reported having all three conditions. Of the 26.1% who visited a gynecologist in the past six months, 27.2% were diagnosed with a reproductive tract infection. Only 32.3% thought that reproductive health services were easily accessible, while 37.8% reported that these services were unavailable and 16.8% did not know if these services existed. Reported barriers to accessing reproductive healthcare were: cost (49.7%), distance or transport (25.4%), fear of mistreatment (7.9%), and other barriers including: security concerns, shame, unavailability of a female doctor, and insufficient provision of services. Of note, 59.7% of respondents had never visited a gynecologist except when pregnant. Although 69.3% of women knew about family planning, only 34.5% were using a family planning method, which is below that reported among the general population in pre-conflict Syria (58.3%) [28]. Consistent with Syrian national statistics, the IUD was the most commonly used method (19.0%) followed by oral contraceptives (8.6%) and the rhythm method (3.5%) [28]. Barriers to contraceptive use included: cost, distance or transportation, unavailability, fear, and personal procrastination.

**Table 2:** Reproductive history, current status, and use of services among 452 Syrian refugee women in Lebanon*

	**N (%) or Mean (±SD)**
Reproductive history	
Age at menarche	15.4 ± 11.1
Age at first pregnancy	19.9 ± 4.4
Number of pregnancies	4.7 ± 3.5
At least one miscarriage	126 (27.9)
At least one abortion (induced)	11 (2.4)
At least one cesarean section	111 (24.6)
At least one child death	80 (17.7)
Current reproductive status	
Pregnant at some point during the conflict	74 (16.4)
Currently pregnant	43 (9.5)
Reported gynecologic issues during conflict:	
Menstrual irregularities	242 (53.5)
Severe pelvic pain/dysmenorrhea	233 (51.6)
Symptoms of reproductive tract infection	241 (53.3)
Perception of RH service availability:	
Available	171 (37.8)
Unavailable	202 (44.7)
Don’t know	76 (16.8)
Perception of RH service accessibility:	
Easily accessible	146 (32.3)
Inaccessible/difficult to access	177 (39.2)
Don’t know	47 (10.4)
Perceived barriers to access (n = 177):	
Price	88 (49.7)
Distance/transport	45 (25.4)
Fear of mistreatment	14 (7.9)
Security concerns	11 (6.2)
Shame/embarrassment	11 (6.2)
Other	8 (4.5)
Use of RH services during past 6 months:	
Visited OB/GYN doctor for any reason	118 (26.1)
Diagnosed with reproductive tract infection	123 (27.2)
Use of Family Planning Method/Contraception:	
None	296 (65.5)
IUD	86 (19.0)
Birth control pill	39 (8.6)
Rhythm method	16 (3.5)
Surgical method	11 (2.4)
Condoms	8 (1.8)
Injection	1 (0.2)

*Numbers may not sum to total due to missing data.

Of the entire sample, 65.9% had ever been pregnant and 74 (16.4%) reported being pregnant at some point during the conflict (Table 3). Of the 74 in the pregnancy subset, 73.0% reported at least one antenatal care visit. Commonly reported barriers to antenatal care were unavailability of a reproductive health clinician (18.9%), cost (9.5%), and distance or transportation (6.8%). Thirty-eight women delivered or aborted during the conflict primarily in a hospital (71.1%) or at home (23.7%). Among these completed pregnancies were: nine preterm births (23.7%), four abortions (10.5%, spontaneous and induced), four low birthweight term infants (10.5%), and one infant death. Complications during labor, delivery, or abortion occurred in 36.8% with hemorrhage being the most commonly reported (29.0%). Of the 33 live births, only 48.5% reported any breastfeeding with inability to breastfeed, illness, and constant displacement cited as reasons for not breastfeeding. Most of the 43 women pregnant at the time of the survey were pregnant upon arrival to Lebanon while 32.6% became pregnant after displacement. Pregnancy problems were reported by 39.5% including: feeling abnormally weak and tired (25.6%), severe abdominal pain (16.3%), vaginal bleeding (9.3%), and fever (4.7%). Of those currently pregnant, 69.8% had at least one antenatal care visit; however, the majority had not accessed antenatal care since coming to Lebanon.

**Table 3:** Characteristics of 74 Syrian refugee women who were pregnant at some time during the conflict*

	**N (%)**
Pregnancy status*	
Currently pregnant	43 (9.5)
Delivered	34 (7.5)
Aborted fetus	4 (0.9)
Primiparous	16 (21.6)
At least one antenatal care visit	54 (73.0)
Pregnancy complications among currently pregnant (n = 43):	
Feeling unusually weak/tired	11 (25.6)
Severe abdominal pain	7 (16.3)
Vaginal bleeding	4 (9.3)
Fever	2 (4.7)
Swelling of hands and face	2 (4.7)
Others (vaginal infection, blurred vision, preeclampsia)	3 (7.0)
Delivery/abortion complications (n = 38):	
Hemorrhage	11 (29.0)
Abnormal vaginal discharge	3 (7.9)
Others (convulsions, fever, hypertension, fetal heart problem, vaginal tearing)	5 (13.2)
Place of delivery/abortion (n = 38):	
Home	9 (23.7)
Hospital	27 (71.1)
Clinic or doctor’s office	2 (5.3)
Birth outcomes among those who delivered (n = 34):	
Preterm birth	9 (26.5)
Low birthweight†	4 (10.5)
Infant death	1 (2.9)

*Numbers may not sum to total due to missing data.

†Infant birthweight not known (n = 3).

### VAW and stress

Almost one-third of women (N = 139, 30.8%) reported exposure to conflict violence and more than a quarter (N = 125, 27.7%) reported exposure to more than one type of conflict violence. Almost all women (95.7%) identified the perpetrator as an armed person and fourteen women (3.1%) disclosed sexual violence perpetrated against them by an armed person in Syria. Of those who experienced violence, 27.7% suffered physical injury and 67.7% suffered psychological difficulties. In bivariate analyses, VAW had a strong positive association with menstrual irregularity (p < 0.001), severe pelvic pain (p < 0.001), RTI symptoms (p < 0.001) among non-pregnant women, and with self-rated health (p = 0.01) among the entire sample. VAW was not associated with accessing obstetric/gynecology services (p = 0.20). In multivariate models, among non-pregnant women, VAW was significantly associated with all gynecologic outcomes, though not with self-rated health (Table 4). Women who experienced violence reported varying help-seeking behaviors: 41.5% did not find a way to cope, 50.8% did not speak with anyone, 24.6% spoke with their husbands, and the remaining spoke with others in the community. Of those exposed to violence, the majority (64.6%) sought no medical care after their experience citing insufficient funds, lack of knowledge, unavailability, embarrassment, and other reasons. Only 9.2% reported accessing any mental health or psychosocial assistance.

**Table 4:** **Exposure to violence and stress score**^**# **^**with various health outcomes among Syrian women**

		**Menstrual irregularity**	**Severe pelvic pain**	**RTI symptoms**	**Self-rated health**
		**(n = 409)**	**(n = 409)**	**(n = 409)**	**(n = 452)**
**Variable:**	**N (%) or mean (range)**	**β (SE)**	**β (SE)**	**β (SE)**	**β (SE)**
Exposure to any conflict violence^†^	139 (30.8%)	0.58 (0.27)*	0.68 (0.26)**	0.53 (0.25)*	0.08 (0.23)
Stress score^‡^	0^#^ (−3.28, 0.94)	0.32 (0.11)**	0.17 (0.11)	0.12 (0.11)	0.30 (0.11)**

*p < 0.05, **p < 0.01, ***p < 0.001

^†^Models are adjusted for: region, age, education, marital status, and anemia; menstrual irregularity model is additionally adjusted for food insecurity; RTI model is additionally adjusted for months in Lebanon; self-rated health is additionally adjusted for food insecurity, hypertension, and cigarette smoking.

^‡^Models are adjusted for: region, age, education, and marital status; model of self-rated health is additionally adjusted for food insecurity, cigarette smoking, and anemia.

^#^Stress score created using Anderson-Rubin method in SPSS which gives a variable with mean 0 and SD 1.

The majority of women in the entire sample (>75%) reported having all seven stress-related symptoms more than usual. Based on principle-component analysis, we included all questions from our original 7-item stress scale in the creation of a “stress score” variable, except for the question about feeling worried or concerned, as it had a low loading in this analysis (0.43). Beating children, as an indicator of stress, had a high loading (0.7) on the stress construct, and was maintained in the stress score variable. Notably, 75.8% of women reported beating their children more than usual. Those who experienced conflict violence had a statistically significantly higher mean stress score than those who did not (p < 0.0001). In bivariate analysis, stress score was found to be associated with menstrual irregularity (p < 0.001), severe pelvic pain (p = 0.02), and RTI symptoms (p = 0.04) among non-pregnant women, and with self-rated health (p < 0.001) in the entire sample. Stress score was not with associated with accessing obstetric/gynecology services (p = 0.81). In multivariate models, non-pregnant women were more likely to have menstrual irregularity with higher levels of stress (p < 0.01). Stress score, however, was not a significant predictor of other gynecologic conditions. Among the entire sample, women with higher levels of stress were more likely to have poor self-rated health (p < 0.01). (Table 4). Stress score was found to mediate the relationship between VAW and self-rated health.

## Discussion

Our findings indicate that Syrian women displaced to Lebanon experience various indicators of poor reproductive health, including: gynecologic conditions, pregnancy and delivery complications, and poor birth outcomes. High reported rates of menstrual irregularity, severe pelvic pain, and vaginal infections align with previous research on gynecologic outcomes in situations of violence or refugee settings [9,16]. Previous research also indicates that poor pregnancy outcomes, including low birthweight and preterm birth, may be related to refugee status, inadequate antenatal care, and economic hardship [9,10,29,30]. All of these factors were present among displaced Syrian women in our sample. In addition to poor reproductive health outcomes, many women rated their health as poor and this was statistically significantly associated with exposure to violence when mediated by stress. Many reported having chronic illnesses, including anemia and hypertension, which may be related to complications surrounding pregnancy and delivery [15,31,32]. Food insecurity, identified among more than half of respondents, may be contributing to menstrual irregularity or to increased anemia.

Exposure to conflict-related violence, abuse, and/or sexual violence was reported by over 30% of women and many women reported multiple types of violence. While several cases of sexual violence perpetrated by armed people in Syria were reported, there may be underreporting of sexual violence due to shame or fear of stigmatization, despite our adherence to WHO guidelines for interviewing survivors of violence [33]. Although WHO has no directly comparable prevalence data on conflict violence or non-partner sexual violence in the Eastern Mediterranean region, we can compare our findings to WHO data in other regions. South East Asia for example has a reported lifetime prevalence of non-partner sexual violence of 4.9%, while our study found a 3.1% prevalence of non-partner sexual violence during the conflict in Syria [15]. Additionally, WHO found a 37.0% prevalence of intimate partner violence in the Eastern Mediterranean region, which suggests that the 30.8% prevalence of conflict violence that we found in our sample may be in addition to violence women are experiencing in the home [15]. Multivariate analyses revealed significant positive associations between exposure to conflict violence and gynecologic conditions (menstrual irregularity, severe pelvic pain, and RTIs), which is consistent with existing literature in both refugee and non-refugee populations, and extends findings focused primarily on intimate partner violence to conflict-related violence. With the recent rise in conflict in the Middle East, it is useful to have a prevalence estimate of conflict violence experienced by women for the purposes of humanitarian response planning.

While the majority of those who experienced conflict violence reported suffering from psychological difficulties, only a small percent (9.0%) accessed mental health services. A previous study found that the majority of Lebanese women exposed to conflict violence and suffering from psychological distress received help from their families while few of them sought medical care [13]. Mental health and psychosomatic conditions reported among survivors of violence could also be a risk factor for poor reproductive health outcomes [14]. Our results suggest a mediating role for stress between exposure to violence and some health outcomes, highlighting the need to include mental health services with other health services for refugees. Many women reported health conditions potentially related to stress, including: nerve issues, depression, unusual pain and fatigue, loss of appetite or sleep, repeated vomiting, and migraines. Outreach to refugee women regarding conflict-related stress could help reduce the burden of mental health and reproductive health conditions, and may attenuate the effect of VAW on reproductive health.

Previous research shows that limited access or delayed entry to antenatal care is a key determinant of pregnancy outcomes in refugee populations, and there are often significant disparities in access to and use of antenatal care among refugee populations compared to non-refugee populations [9]. Our study supports this finding and goes on to identify perceived barriers to access among a Middle Eastern population. The majority of Syrian refugee women had never visited an obstetrician-gynecologist except for pregnancy care, indicating low baseline rates of gynecological exams. While costs and long distances were the primary barriers reported, one unique barrier was lack of availability of a female gynecologist (as many women requested to be seen only by a female doctor). These findings indicate the need to increase awareness of the importance of antenatal care and increase availability of female physicians to provide these services in a culturally sensitive manner.

This study has several limitations. Although we selected a proportionally representative sample, results cannot be generalized to all Syrian refugee women in Lebanon, particularly to those who may not have access to clinics. Since this is an ongoing conflict setting and the number of Syrian refugees is increasing over time, it is possible that newer refugees may have different characteristics (e.g., age, SES, ethnic/religious affiliations, etc.) or potentially different needs. However, this study provides much-needed baseline data to assess the rapidly evolving situation. In addition, we relied on self-report, which may be subject to under- or over-reporting and is of particular concern for key reproductive health outcomes and sexual violence. Although we tried to ensure privacy, revealing such personal information during an interview may still be sensitive. Also, sexual violence questions were not asked using “event-based” items (e.g. forced to engage in a sexual act when participant did not want to), which may increase under-reporting [34]. Finally, the survey location at health care centers poses a limitation on generalizability and prevalence estimates, as women presenting to these clinics may differ from the general population with respect to health status, behaviors, and knowledge of health services. Due to the fast-moving nature of this conflict setting, there is no data available on the total number of female Syrian refugees attending clinics in Lebanon during the time period of our study. UNHCR reports that 248,000 Syrian refugees have been assisted with primary healthcare to date (August 2013), indicating that over half of Syrian refugees have not attended a health clinic (though this estimate is not disaggregated by gender) [4]. Despite limitations, this study provides important information about a vulnerable population that continues to grow as the conflict shows no signs of abating. In addition, information gained from this study may assist in planning for future humanitarian crises involving large numbers of displaced women and children.

## Conclusions

This study contributes to understanding of reproductive health needs among conflict-affected women and, in particular, among refugee women in the Middle East. The significant relationship between violence and reproductive health additionally indicates the need to integrate mental health and reproductive health services for refugee women. While some services are in place for survivors of violence, our findings indicate a need for greater provision and awareness of existing services, while addressing barriers preventing displaced populations from accessing them.

## Competing interests

The authors declare that they have no competing interests.

## Authors’ contributions

ARM, JU, and ASE designed study, conducted literature search and wrote manuscript. ARM conducted study and analyzed data in consultation with JU and ASE. JG contributed to literature search, manuscript, and interpretation of results. All authors read and approved the final manuscript.

## Pre-publication history

The pre-publication history for this paper can be accessed here:

http://www.biomedcentral.com/1472-6874/14/25/prepub
